# In vitro generation of transplantable insulin-producing cells from canine adipose-derived mesenchymal stem cells

**DOI:** 10.1038/s41598-022-13114-3

**Published:** 2022-06-01

**Authors:** Quynh Dang Le, Watchareewan Rodprasert, Suryo Kuncorojakti, Prasit Pavasant, Thanaphum Osathanon, Chenphop Sawangmake

**Affiliations:** 1 International Program of Veterinary Science and Technology, Faculty of Veterinary Science, Chulalongkorn University, Bangkok, Thailand; 2 Veterinary Stem Cell and Bioengineering Innovation Center (VSCBIC), Veterinary Pharmacology and Stem Cell Research Laboratory, Faculty of Veterinary Science, Chulalongkorn University, Bangkok, Thailand; 3 Department of Veterinary Science, Faculty of Veterinary Medicine, Universitas Airlangga, Surabaya, East Java Indonesia; 4 Veterinary Stem Cell and Bioengineering Research Unit, Faculty of Veterinary Science, Chulalongkorn University, Bangkok, Thailand; 5 Department of Pharmacology, Faculty of Veterinary Science, Chulalongkorn University, Bangkok, Thailand; 6 Department of Anatomy, Faculty of Dentistry, Chulalongkorn University, Bangkok, Thailand; 7 Dental Stem Cell Biology Research Unit, Faculty of Dentistry, Chulalongkorn University, Bangkok, Thailand; 8 Center of Excellence in Regenerative Dentistry (CERD), Faculty of Dentistry, Chulalongkorn University, Bangkok, Thailand

**Keywords:** Cell signalling, Stem cells, Adult stem cells, Mesenchymal stem cells, Regeneration, Stem-cell differentiation, Immunochemistry, RNA

## Abstract

Canine mesenchymal stem cells (cMSCs) have potential applications for regenerative therapy, including the generation of insulin-producing cells (IPCs) for studying and treating diabetes. In this study, we established a useful protocol for generating IPCs from canine adipose mesenchymal stem cells (cAD-MSCs). Subsequently, in vitro preservation of pluronic F127-coated alginate (ALGPA)-encapsulated cAD-MSC-derived IPCs was performed to verify ready-to-use IPCs. IPCs were induced from cAD-MSCs with the modulated three-stepwise protocol. The first step of definitive endoderm (DE) induction showed that the cooperation of Chir99021 and Activin A created the effective production of *Sox17*-expressed DE cells. The second step for pancreatic endocrine (PE) progenitor induction from DE indicated that the treatment with taurine, retinoic acid, FGF2, EGF, TGFβ inhibitor, dorsomorphin, nicotinamide, and DAPT showed the significant upregulation of the pancreatic endocrine precursor markers *Pdx1* and *Ngn3*. The last step of IPC production, the combination of taurine, nicotinamide, Glp-1, forskolin, PI3K inhibitor, and TGFβ inhibitor, yielded efficiently functional IPCs from PE precursors. Afterward, the maintenance of ALGPA-encapsulated cAD-MSC-derived IPCs with VSCBIC-1, a specialized medium, enhanced IPC properties. Conclusion, the modulated three-stepwise protocol generates the functional IPCs. Together, the encapsulation of cAD-MSC-derived IPCs and the cultivation with VSCBIC-1 enrich the maturation of generated IPCs.

## Introduction

Diabetes mellitus (DM) is a complex metabolic disorder characterized by a chronic presence of hyperglycemia and glycosuria as the results of insulin deficiency or impaired insulin response to target tissues^1–3^. DM is one of the common endocrine diseases diagnosed in the canine family besides human beings^4^. An epidemiological study in the United States reported a 32 percent increase in canine diabetes between 2006 and 2011, and the data kept on rising by 47.7 percent from 2011 to 2016^5^. By pathophysiological diagnosis, canine DM has been mostly concerned with beta cell deficiency by latent autoimmunity, which is considered as similar with human type 1 DM (T1DM)^6,7^. Insulin therapy has been clinically well-established to manage T1DM in both dogs and humans, however, adverse events and disadvantages have also been periodically reported^8–10^. In 2000, a trial of islet transplantation was performed successfully according to “Edmonton protocol”, thus this method introduced as an alternative approach for treating long-term hyperglycemia with insulin independence^11–13^. Although pancreatic islet transplantation can surmount the impediments of insulin therapy, the lack of donor islet source and the immune reactivity of recipients exist as two main obstacles of this method^11,12,14^. To solve the restrictions of the Edmonton protocol, the tendency of regenerative medicine production in which insulin-producing cells (IPCs) derived from stem cells has been a promising candidate^12,15^.

Current in vitro IPCs are generated by embryonic stem cells (ESCs), induced pluripotent stem cells (iPSCs) and mesenchymal stem cells (MSCs). Nevertheless, ESCs have encountered ethical issues, while reprogramming of iPSCs can cause teratoma formation and side effects of pluripotency-induced viral transgenes might be unsafe for clinical applications^16^. Meanwhile, MSCs possess immune-privileged and highly plastic abilities, this allows MSCs to be a wonderful and safe choice for IPC generation^16^. The capacity of human MSCs (hMSCs) differentiated into IPCs as well as their clinical accomplishment has been shown in many previous studies^17–20^. Although a minority of research on IPCs has originated from canine MSCs (cMSCs)^21–23^, these cMSC-derived IPCs are still functionally inadequate and morphologically circumscribed. To fabricate the effective cMSC-derived IPCs, it is essential to advance the current differentiation protocols. In types of cMSCs, canine adipose-derived MSCs (cAD-MSCs) are an accessible candidate and possess the potency for IPC differentiation^22,23^. Therefore, this study focused on establishing a protocol for cAD-MSCs induction toward mature IPCs in vitro*.* Moreover, preservation of cAD-MSC-derived IPCs, which were encapsulated in alginate gel and pluronic acid (ALGPA) following our previous study^24^, would also be investigated. This knowledge will aid the fundamental insights for in vitro cAD-MSC-derived IPC generation and eventually for in vivo transplantation study.

## Results

### Isolation and characterization of cAD-MSCs

**Figure 1:**
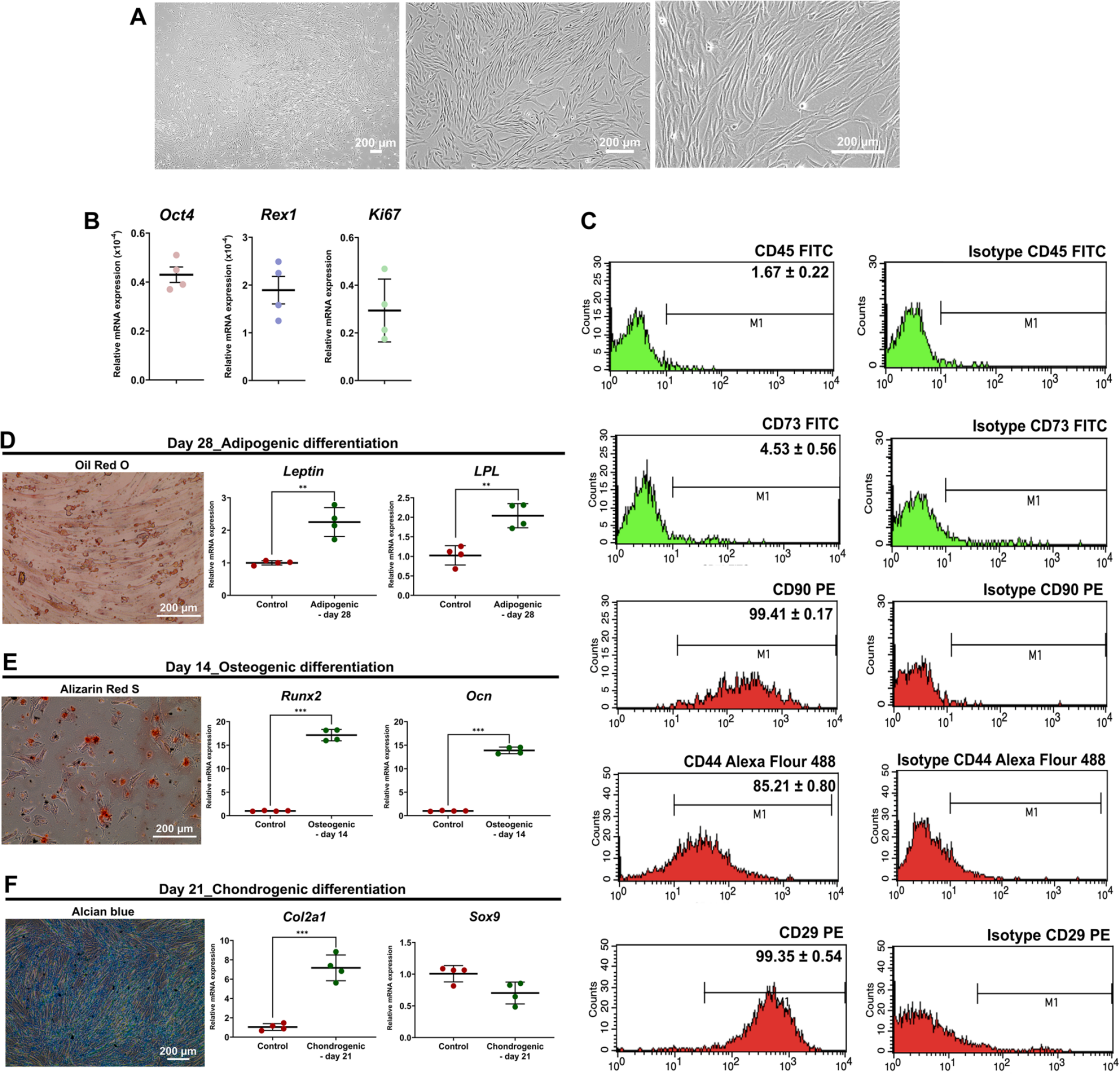
cAD-MSC characteristics. Morphological appearances of cAD-MSCs were observed under a light microscope with magnification of 4X, 20X, and 40X (**A**). The mRNA expression of stemness markers (*Oct4*, and *Rex1*) and a proliferation marker (*Ki67*) were analyzed by RT-qPCR (**B**), normalized with the reference gene (*Gapdh*). Expression of surface markers exhibiting MSC property were revealed using flow cytometry (**C**). Adipogenic differentiation potential at day 28 post-induction was stained with Oil Red O, and adipogenic related-mRNA expression was determined (**D**). Osteogenesis was confirmed using Alizarin Red S staining at day 14 post-induction, and the expression of osteogenic mRNA markers were assessed (**E**). Chondrogenic differentiation potential at day 21 post-induction was demonstrated by Alcian blue staining, and chondrogenic mRNA markers were determined (**F**). The expression of mRNA genes related to multilineage differentiation was normalized with the reference gene and the undifferentiation control. Bars indicates the significant differences (**, *p* value <0.01, ***, *p* value <0.001).

cAD-MSCs showed adherent-dependent and fibroblast-like cells on the 2 dimensions (2D) culture (Fig. 1A). The mRNA expression of stemness markers (*Oct4* and *Rex1*) and proliferative marker (*Ki67*) were detected by RT-qPCR (Fig. 1B). In addition, MSC-related surface markers using flow cytometry revealed the strong expression of CD29 and CD90, moderate expression of CD44, low expression of CD73, but absent expression of CD45 (Fig. 1C).

Moreover, in vitro multi-differentiation potential toward adipogenic, osteogenic, and chondrogenic lineages was observed. cAD-MSCs dramatically upregulated adipogenic-related genes (*Leptin* and *LPL*) while the production of lipid droplets was detected by Oil Red O staining (Fig. 1D). Regarding osteogenic differentiation, the substantial upregulation of *Runx2* and *Ocn* was indicated upon exposed cells in osteogenic induction medium and osteocyte-produced calcium deposits were recognized by Alizarin Red S staining (Fig. 1E). For chondrogenic differentiation, the upregulation of the *Col2a1* gene was significantly disclosed, and glycosaminoglycan accumulation was stained with Alcian blue (Fig. 1F).

Thus, the isolated cAD-MSCs show homogeneous appearance and the differentiation potential toward other cell lineages.

### Cooperation of GS3K inhibitor Chir99021 with Activin A enhances definitive endoderm formation from cAD-MSCs

**Figure 2:**
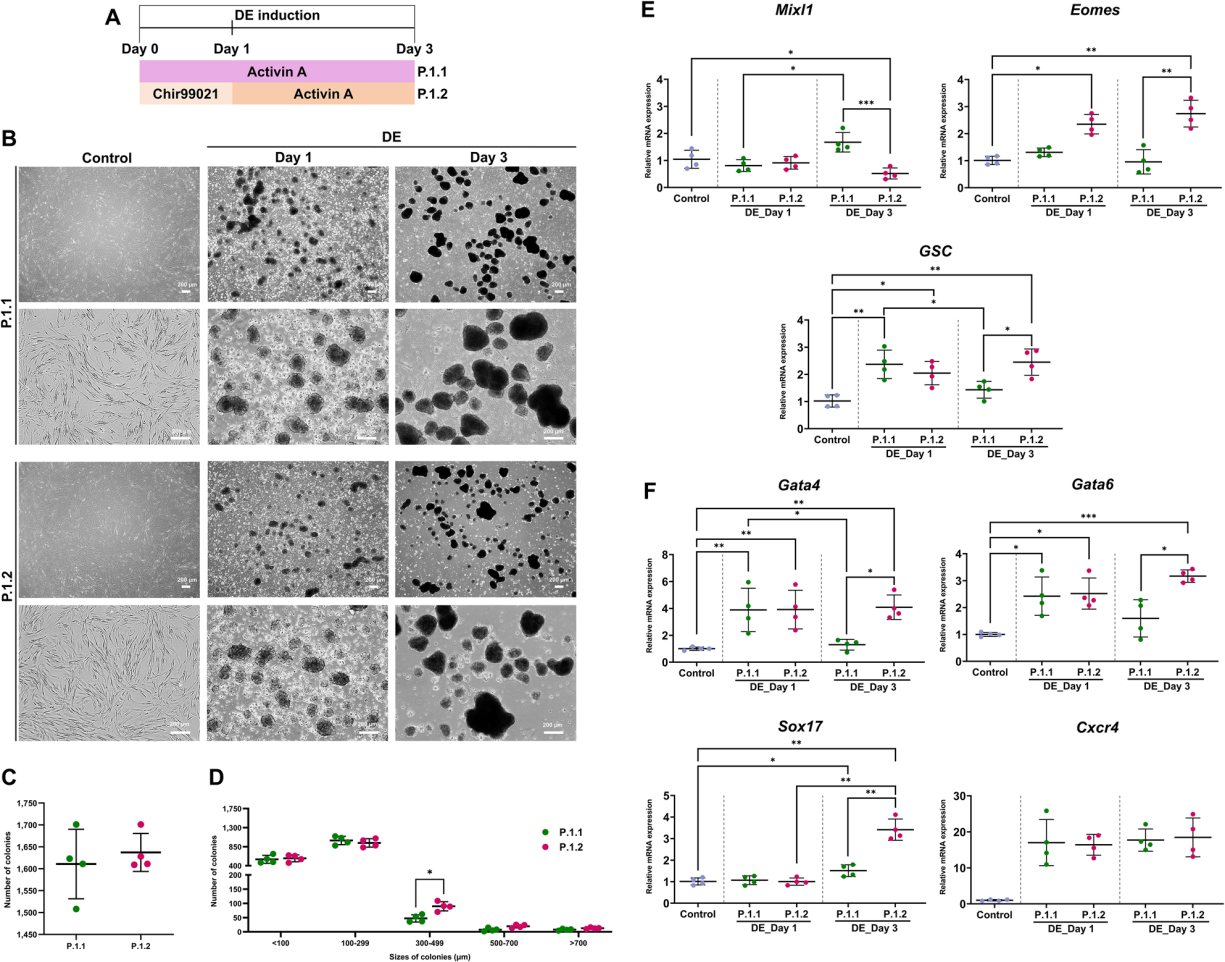
Generation of cAD-MSC-derived DE cells. The diagrams of two protocols used for the induction of cAD-MSC derived DE cells are shown in (**A**). Differentiation of morphological appearances of cAD-MSCs toward DE cells was observed at day 1, and 3 (**B**). The total colony number (**C**) and the distribution of colony sizes (**D**) were evaluated. The expression of mRNA markers relating to mesendoderm status (**E**), and definitive endoderm status (**F**) was analyzed by RT-qPCR at day 1, and 3 post-induction. Relative mRNA expression was normalized with the reference gene, and the undifferentiation control. Bars indicates the significant differences (*, *p* value < 0.05; **, *p* value < 0.01; ***, *p* value < 0.001).

First, formation of definitive endoderm (DE), a germ cell layer, is an essential initiation step for giving rise to pancreatic cells^25,26^. DE were generated from cAD-MSCs using DE induction media supplemented with Activin A alone for 72 h (protocol 1.1; P.1.1) or Chir99021 for first 24 h and following with Activin A for 48 h (protocol 1.2; P.1.2) (Fig. 2A). Small three-dimension (3D) clusters were formed from dissociated cAD-MSCs cultured in suspension for 24 h, then size and density of colonies were increased (Fig. 2B). On day 3, the total colony counts (medium) were 1610.75 and 1637.25 colonies per batch (1 × 10^6^ seeding cells) in P.1.1 and P.1.2 groups, respectively (Fig. 2C). Interestingly, the distribution of size-based colonies showed that the small-to-medium colony size (100–299 μm) occupied the most population in both protocols, and P.1.2 yielded significantly more medium-size colonies (300–499 μm) than P.1.1 (Fig. 2D).

Levels of mRNA expression related to mesendoderm (ME) and DE were analyzed and compared to undifferentiated cells. The ME-related markers (*Eomes* and *GSC*) in P.1.2 showed the upregulated expression in a time-dependent manner (Fig. 2E). In contrast, the expression of *Mixl1* was upregulated in P.1.1, while P.1.2 was downregulated. For the DE-related markers, both protocols upregulated all genes comparing with undifferentiated control (Fig. 2F). However, P.1.2 was able to increase the expression of *Gata4*, *Gata6*, and *Sox17* on the last day of DE induction.

Generally, the result revealed that the cooperation of Chir99021 and Activin A is effectively on the generation of DE from cAD-MSCs with small-size and the crucial DE-related markers.

### Combination of signaling modulators systematically generates pancreatic endocrine precursors

**Figure 3:**
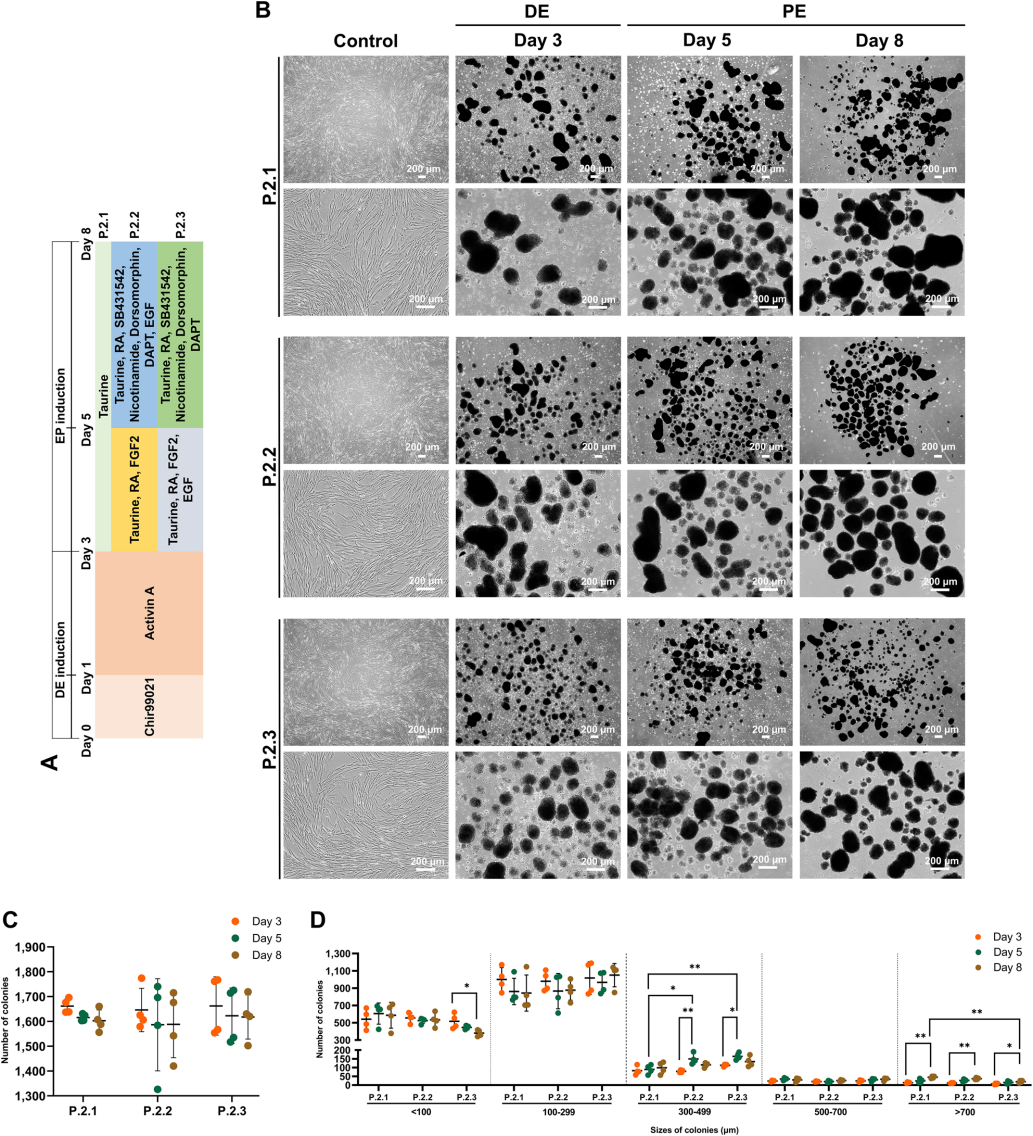
Generation of cAD-MSC-derived PE cells. The diagrams of three protocols used for the induction of cAD-MSC-derived PE cells are shown in (**A**). Differentiation of morphological appearances of cAD-MSCs toward PE cells was observed at day 3, 5 and 8 (**B**). The total colony number (**C**) and the distribution of colony sizes (**D**) were evaluated. Bars indicates the significant differences (*, *p* value < 0.05; **, *p* value < 0.01).

Pancreatic endocrine (PE) stage is the vital second stage on pancreatic development process according to its important roles in cell fate modulation on DE cell toward pancreatic cell types^27,28^. In this part, three established protocols were explored; protocol 2.1 (P.2.1), protocol 2.2 (P.2.2), and protocol 2.3 (P.2.3) (Fig. 3A). All protocols exposed the 3D floating colonies which became bigger and denser along the culture period (Fig. 3B). All protocols showed the similar trend of total colony count, however, there were gently decreasing by the induction day (Fig. 3C). For the sized-based colony number, the number of colonies was mostly found in 100–299 μm, nevertheless, the large-size colonies were slightly increasing during the induction period (Fig. 3D).

**Figure 4:**
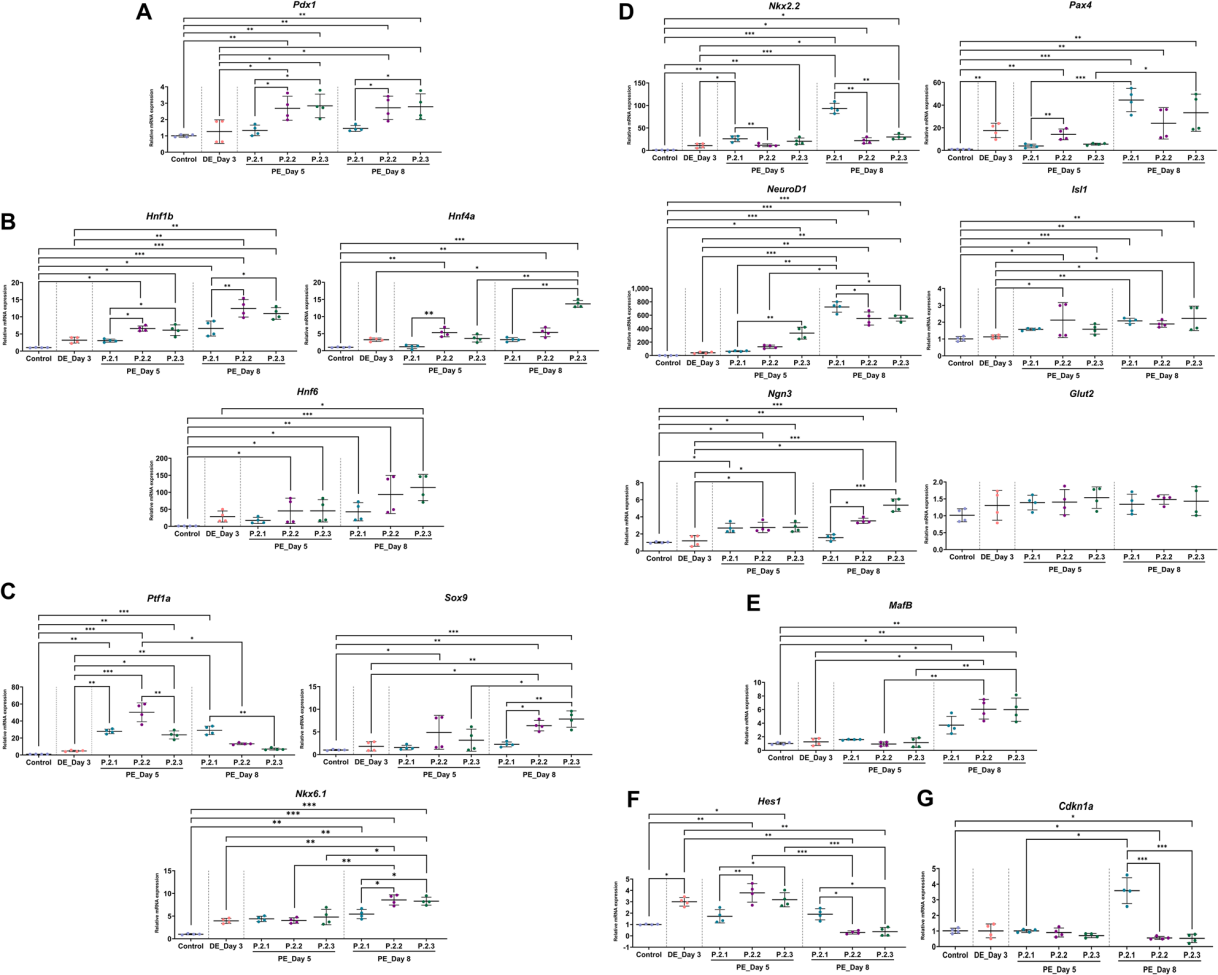
PE-related marker expression. The expression of mRNA markers relating to pancreatic endoderm (**A**), primitive gut and posterior foregut (**B**), pancreatic multipotent progenitor (**C**), endocrine precursors (**D**), immature pancreatic endocrine (**E**), caNotch pathway target gene (**F**), and a cell cycle regulator (**G**) was analyzed by RT-qPCR at day 3, 5 and 8 post-induction. Relative mRNA expression was normalized with the reference gene, and the undifferentiation control. Bars indicates the significant differences (*, *p* value < 0.05; **, *p* value < 0.01; ***, *p* value < 0.001).

Except for the *Glut2* gene, the expression of other mRNA markers relating to the development of PE precursors exhibited significant differences among the protocols. Compared to the undifferentiated cells and DE cells on day 3, the expression of pancreatic endoderm marker (*Pdx1*) was upregulated in groups treated with P.2.2 and P.2.3 on day 5 and day 8, while there was not detected in P.2.1 (Fig. 4A). The expression of *Hnf1b*, *Hnf4a*, and *Hnf6*, known as primitive gut and posterior foregut markers, was upregulated on day 5 and day 8 post-induction in P.2.2 and P.2.3 (Fig. 4B). The multipotent pancreatic progenitor (MPP) marker (*Ptf1a*) was upregulated on day 5 in three protocols, however, the trend of *Ptf1a* downregulation was found in P.2.2 and P.2.3 on day 8 (Fig. 4C). On other hand, P.2.2 and P.2.3 exhibited the upregulation trend of the other MPP markers (*Sox9* and *Nkx6.1*) on the last day of PE induction (Fig. 4C). All of the protocols showed the increased expression of the PE-related mRNA markers such as *Nkx2.2*, *Pax4*, *Ngn3*, *NeuroD1*, and *Isl1* on day 8, compared to undifferentiated cells (Fig. 4D). On day 8, it is noticed that the highest expression of *Nkx2.2* and *NeuroD1* was found in P.2.1, whereas the greatest expression of *Ngn3*, a master key for endocrine specification, was found in P.2.3. In addition, the immature pancreatic endocrine marker, *MafB*, was tremendously upregulated in P.2.2 and P.2.3 on day 8 compared to the undifferentiated cells (Fig. 4E). Moreover, P.2.2 and P.2.3 displayed the significant downregulation of *Hes1*, Notch target gene, on the last day of PE induction (Fig. 4F). Besides, compared with cAD-MSCs, P.2.1 increased the expression of *Cdkn1a*, a cell cycle regulator, on day 8, while the decreased expression was observed in P.2.2 and P.2.3 (Fig. 4G).

Regarding all the results, the new combination of signaling modulators supports the PE development. Importantly, the obtained PE cells from P.2.3 showed the greatest PE-related mRNA expression.

### Combination of forskolin, PI3K inhibitor, and TGFβ inhibitor enriches the functional maturation of IPCs

**Figure 5:**
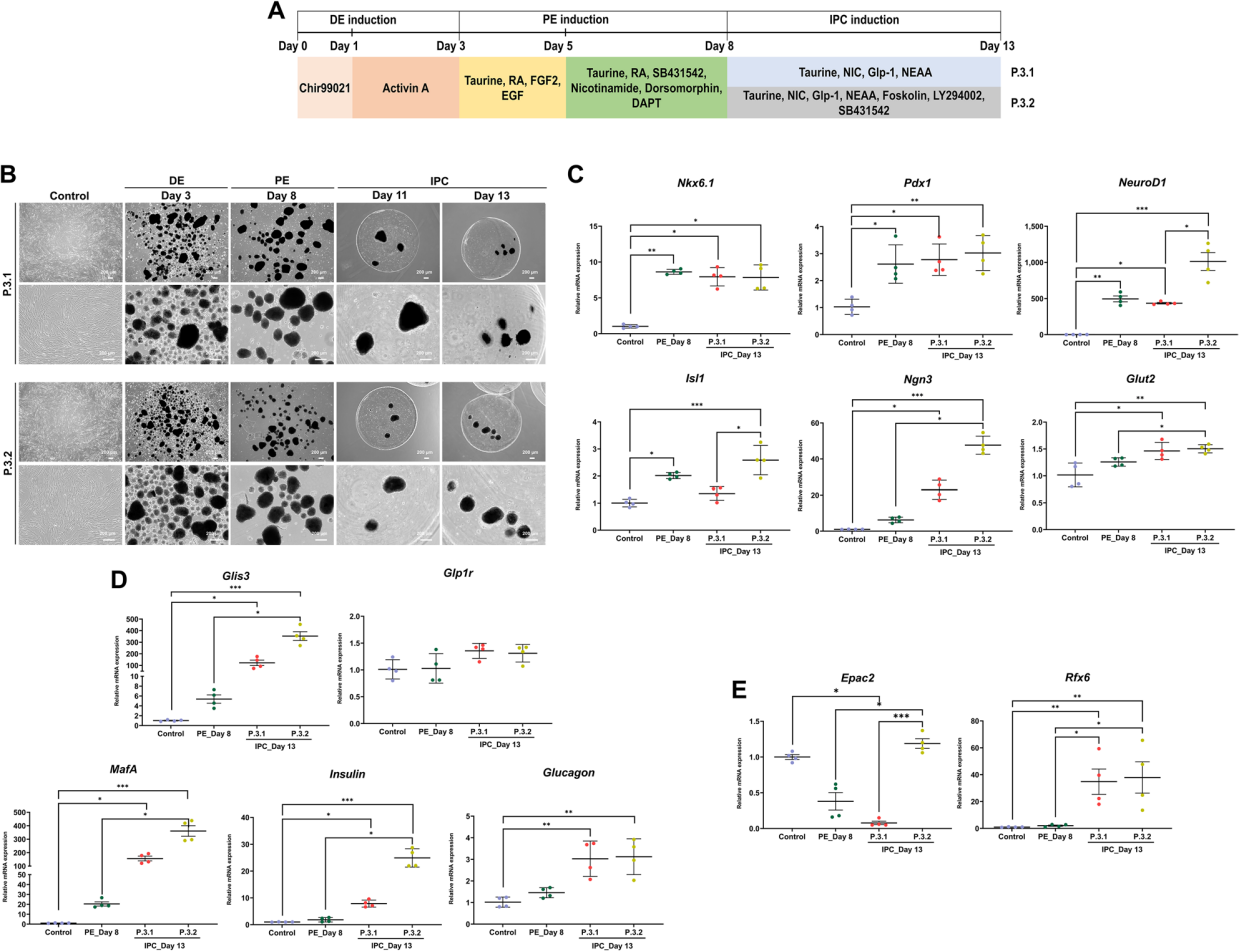
Generation of cAD-MSC-derived IPCs. The diagrams of two protocols used for the induction of cAD-MSC derived IPCs are shown in (**A**). Differentiation morphological appearances of cAD-MSCs toward IPCs were observed at day 8 and 13 post-induction (**B**). The expression of mRNA markers relating to PE precursors (**C**), mature endocrine progenitors (**D**), hormone release-related markers (**E**). Relative mRNA expression was normalized with the reference gene, and the undifferentiation control. Bars indicates the significant differences (*, *p* value < 0.05; **, *p* value < 0.01; ***, *p* value < 0.001).

Last induction, two cocktail media were established to effectively induce functional IPCs from PE precursors; formulas of protocol 3.1 (P.3.1) and protocol 3.2 (P.3.2) are defined in Fig. 5A. Therefore, PE colonies on day 8 were encapsulated in alginate gels, then the encapsulated PE were induced toward IPCs. Both protocols were still showing a 3D colony morphology during IPC induction period (Fig. 5B).

Except for *Nkx6.1* gene, the mRNA expression of PE progenitor-related markers (*Pdx1*, *NeuroD1*, *Isl1*, *Ngn3*, and *Glut2*) was upregulated in P.3.2, compared to undifferentiated cells and PE cells on day 8 (Fig. 5C). Conversely, the downregulation trend of *Nkx6.1*, *NeuroD1*, *Isl1* was found in P.3.1 compared to PE cells (Fig. 5C). The expression of mature endocrine markers, including *Glis3*, *MafA*, *Insulin*, and *Glucagon*, was upregulated in both of protocols, compared with those from undifferentiation cells and PE cells (Fig. 5D). However, the highest expression of mature endocrine markers was explored in P.3.2. Furthermore, P.3.2 showed the upregulation of hormone release-related markers (*Epac2* and *Rfx6*), compared to undifferentiated cells and PE cells (Fig. 5E). Although P.3.1 showed the trend of increasing *Rfx6* expression, the downregulation of *Epac2* was found in this protocol (Fig. 5E).

**Figure 6:**
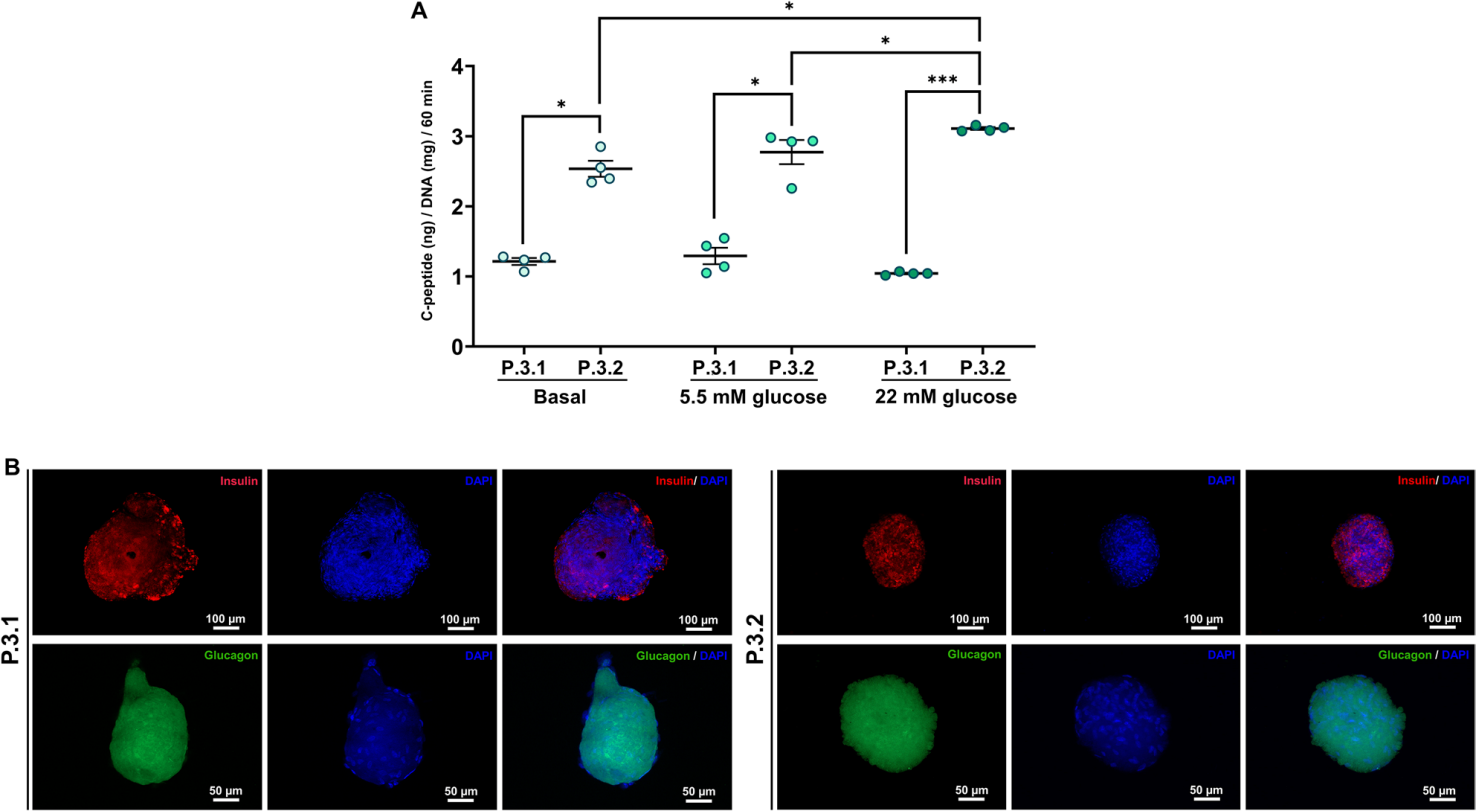
Functionality of cAD-MSC-derived IPCs. C-peptide secretion was determined by glucose-stimulated C-peptide secretion (GSCS) assay (**A**). The expression of Insulin and Glucagon by cAD-IPCs was detected by immunocytochemistry on day 13 by immunocytochemistry and observed under fuorescent microscope ZEISS Apotome.2 (Carl Zeiss, Germany) incorporated with Axio Observer Z1 and ZEN pro sofware (ZEISS International, Germany) at 10X (Insulin) and 20X (Glucagon) (**B**). Bars indicates the significant differences (*, *p* value < 0.05; ***, *p* value < 0.001).

At day 13 of IPC induction, cAD-MSC-derived IPCs in both P.3.1 and P.3.2 were further evaluated the functional potential regarding the production of C-peptide upon glucose stimulation at two different concentrations, 5.5 mM and 22 mM. The findings showed that IPCs in both the protocols yield C-peptide under a basal condition, the higher production of C-peptide was found in P.3.2 (Fig. 6A). Moreover, IPCs from P.3.2 secreted C-peptide in concentration-dependent manner upon high (22 mM) glucose stimulation, compared to those from basal control and P.3.1 (Fig. 6A). In addition, immunocytochemistry staining was employed to confirm the expression of the crucial pancreatic islet-related hormones, Insulin and Glucagon. The result suggested that the expression of these proteins was observed on cAD-MSC-derived IPCs in both protocols on day 13 post-induction (Fig. 6B).

Taken together, the results suggested that P.3.2 which used forskolin, PI3K inhibitor, and TGFβ inhibitor could have more positive effects on IPCs’ maturation from cAD-MSC-derived PE cells.

### Nourishment of specialized medium, VSCBIC-1, on ALGPA-encapsulated IPC’s sustenance

**Figure 7:**
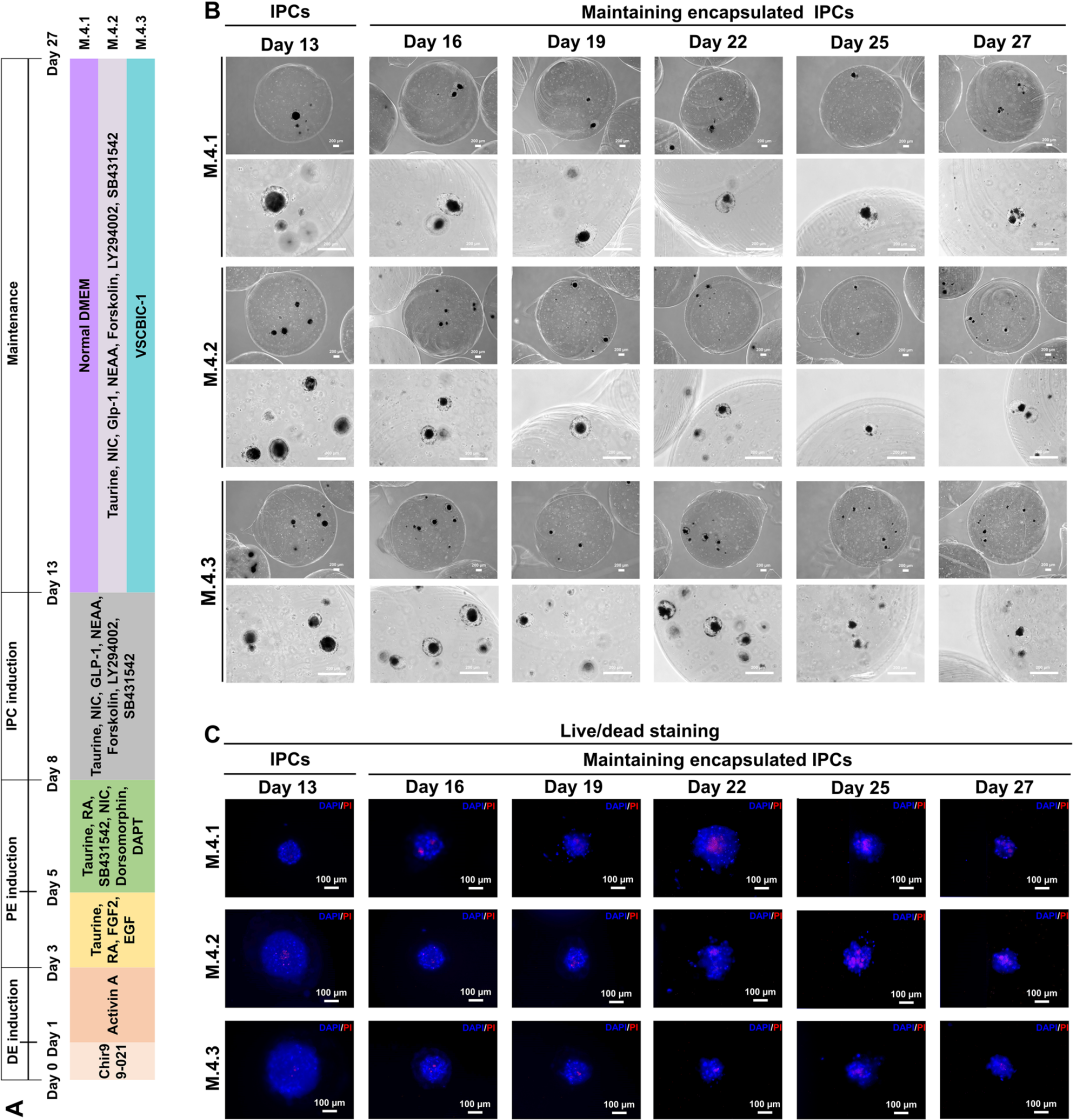
Morphological and viability evaluation of ALGPA-encapsulated cAD-MSCs-derived IPCs. The cAD-MSC-derived IPC induction diagram and media maintaining IPCs encapsulated in ALGPA are shown in (**A**). ALGPA-encapsulated cAD-MSCs-derived IPCs’ morphological appearances were observed at day 13, 16, 19, 22, 25, and 27 post induction (**B**). The viability evaluation of encapsulated cAD-MSC-derived IPCs was determined by live/dead staining (**C**).

**Figure 8:**
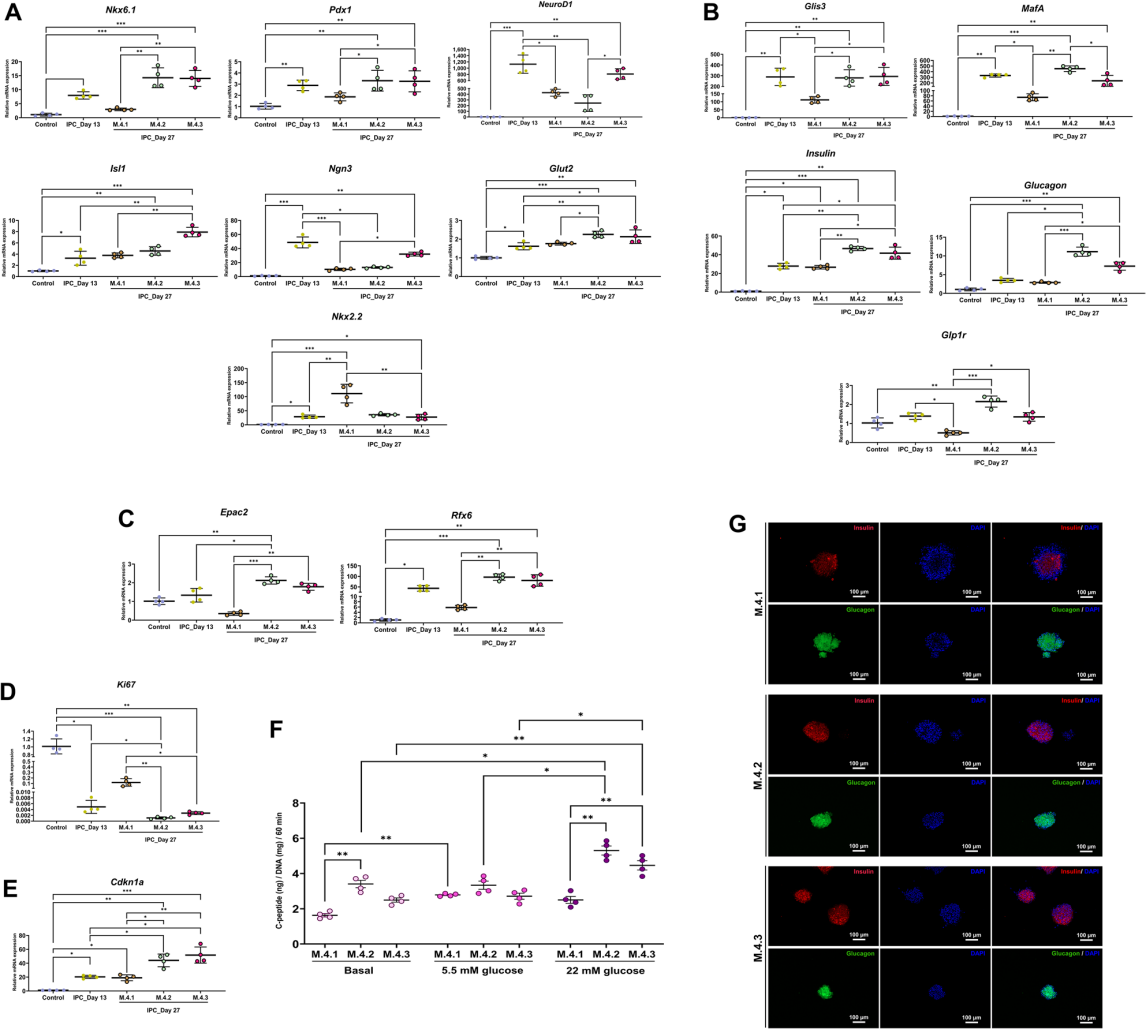
Sustainable functionality of VSCBIC-1 on ALGPA-encapsulated cAD-MSCs-derived IPC maintenance. The expression of mRNA markers relating to PE precursors (**A**), mature endocrine progenitors (**B**), hormone-releasing markers (**C**), a proliferation marker (**D**), and a cell cycle regulator (**E**) was analyzed by RT-qPCR at day 27 post-induction. C-peptide secretion was determined by glucose-stimulated C-peptide secretion (GSCS) assay (**F**). The expression of Insulin and Glucagon by cAD-IPCs was detected on day 27 by immunocytochemistry and observed under fuorescent microscope ZEISS Apotome.2 (Carl Zeiss, Germany) incorporated with Axio Observer Z1 and ZEN pro sofware (ZEISS International, Germany) (**G**). Relative mRNA expression was normalized with the reference gene, and the undifferentiation control. Bars indicates the significant differences (*, *p* value < 0.05; **, *p* value < 0.01; ***, *p* value < 0.001).

It is essential to generate ready-to-use (RTU) IPCs for in vivo application. Maintenance of ALGPA-encapsulated cAD-MSC-derived IPCs in vitro is required. Hence, three different media were chosen to maintain the ALGPA-encapsulated cAD-MSC-derived IPCs in vitro after 13 days of IPC induction process (Fig. 7A). Medium 4.1 (M.4.1) is normal DMEM, medium 4.2 (M.4.2) is an IPC induction medium (P.3.2), and medium 4.3 (M.4.3) is VSCBIC-1, our specialized medium^29^. PE progenitors on day 8 would be double encapsulated in ALGPA instead of only alginate. Morphological changes were observed for 2 weeks after 13 days of IPC induction process (Fig. 7B). Damaged IPCs were not found in ALGPA, but the colonies became smaller in all three media. All maintenance media showed the viability of ALGPA-encapsulated cAD-MSC-derived-IPCs until day 27 by live/death staining (Fig. 7C). The mass of non-viable cells was detected in all three media along the maintenance period. In addition, cAD-MSC-derived IPCs on day 27 in M.4.2 and M.4.3 exhibited the upregulation trend of PE mRNA markers (*Nkx6.1*, *Pdx1*, *Isl1*, and *Glut2*) compared IPCs from day 13 and IPCs from M.4.1 (Fig. 8A). Although the decreasing of *Ngn3* and *NeuroD1* was found in all media compared with IPCs on day 13, M.4.3 showed the insignificant decline (Fig. 8A). Interestingly, the increasing of *Nkx2.2* was showed in M.4.1 (Fig. 8A). In other hand, the upregualtion of mature endocrine markers (*Glis3*, *MafA*, *Insulin*, *Glucagon*, and *Glp1r*) and hormone release-related markers (*Epac2*, and *Rfx6*) was explored in M.4.2 and M.4.3 compared with undifferentiated cells, IPCs from day 13, and IPCs from M.4.1 (Fig. 8B,C). Although the proliferation marker, *Ki67*, of IPCs was lower expressed in M.4.2 and M.4.3 than M.4.1, the upregulation of a cell cycle regulator, *Cdkn1a,* was found in M.4.2 and M.4.3 groups (Fig. 8D,E).

The functional property was evaluated by the level of C-peptide secretion (Fig. 8F). The result showed the trend of concentration-dependent response upon 22 mM glucose stimulation in M.4.2 and M.4.3 groups, while M.4.1 exhibited the releasing of C-peptide level response to 5.5 mM glucose stimulation. Additionally, the protein expression of Insulin and Glucagon was done using immunocytochemical staining. The result confirmed that both hormones were detected in all maintenance media (Fig. 8G).

According to the results, the utilization of VSCBIC-1 reveals the preservation effect of ALGPA-encapsulated cAD-MSC-derived IPCs together with the maturation of produced IPCs.

## Discussion

The concept of stem cell therapy holds gigantic promise for treating diabetes^30^, MSCs provide an auspicious platform to produce clinically applicable IPCs^31–37^. In this study, cAD-MSC induction protocol toward mature IPCs and the enriched medium for preserving AGLPA-encapsulated cAD-MSC-derived IPCs were established to produce RTU IPCs. The cAD-MSCs were isolated, cultured, and expanded following our previous report^38^. These isolated cAD-MSCs were then defined following the International Society for Cellular Therapy (ISCT)^39^, they presented fibroblast-like cell sharp, adherent to culture plastic. In addition, pluripotent markers *Oct4* and *Rex1* were expressed, which reflects the multipotent and proliferative properties^40,41^. Furthermore, MSCs were required to exhibit CD73, CD90, CD44, and CD29 surface markers, and lack expression of CD45 marker^39^. Similar to our result, other previous studies also illustrated low expression of CD73 on various types of cMSCs, even on MSCs of some other animals^42,43^. It is noted that CD73 expression could be different among various sources and species. Moreover, the isolated cells showed capacity toward adipogenic, chondrogenic, osteogenic differentiation as same as previous studies^41,44–48^. All these evidences reflect the potential and homogeneity of the isolated cAD-MSCs.

Currently, various differentiation protocols of IPCs were published^49–54^. The strategies of these protocols were established on ESCs and iPSCs. Although ESC-derived IPCs have achieved positive outcomes of insulin-secreting regulation in clinical trials^55,56^, ESCs are acrimoniously encountering with ethical issues^55–57^. Alternatively, iPSCs, which are generated from somatic cells, are promoted due to avoiding from the ethics and resolving MSC’s drawbacks^58^. Nevertheless, iPSCs need to overcome other challenges, including tumorigenicity, and genetic instability^59,60^. In 2020, Adrian et al. conducted the generation of good manufacturing practice (GMP)-grade iPSC-MSCs for using in clinical trials, which overcame the risk of teratoma formation by a filtration process^61^. However, almost cell reprogramming is often made using viral vectors which is a serious concern^62–64^, and the simpler and cost-effective method is required for preventing teratoma formation^65^. Compared to ESCs and iPSCs, adult tissue-MSCs are inherently restricted by imperfect capacity of proliferation and weakened differentiation capacity with prolonged culture and high passage number^66,67^, but MSCs are exempted from the ethical limitations and iPSC’s restrictions. Furthermore, MSCs are widely used in clinical trials^68^, thus MSCs are offered as a good candidate for IPC generation^16^. The MSC-based protocols mainly comprise of three differentiation stages^16,38,69–71^. The different stages for MSCs are DE, PE progenitors, and IPC maturation. Although IPCs could be produced from cAD-MSCs with the simple three-stepwise protocol^69,70^, their function is still hamperred^38^. Here, we illustrated that the modified three-stepwise protocol could improve the in vitro functional IPC differentiation from cAD-MSCs.

As the first step of IPC-derived MSCs, DE formation is a prerequisite for generating efficient pancreatic lineage from MSCs^72^. In previous studies, Activin A and Chir99021 were known as the potential small molecules on DE development^38,69,70,73^. Although the independent utilization of Chir99021 and Activin A could induce DE from stem cells^74–77^, the cooperation of Chir99021 and Activin A could optimize for inducing DE cells^76,77^. Here, Chir99021, a strong indirect activator of the canonical Wnt-pathway via inhibition of GSK3β signaling pathway, allows MSCs differentiate into ME, a mesendoderm lineage^76,77^. Subsequently, DE is induced from ME by treating with Activin A which is known as an endogenous noggin^25,26,73,78^. The cascade of ME specification before DE is considered as physiologically relevant^79^. Previous studies found that at ME stage, the cells expressed *Mixl1*, *Eomes*, and *GSC* and at DE stage expressed *Gata4/6*, *Sox17*, and *Cxcr4*, meanwhile the roles of these markers are modulating the cell’s decision deriving to ME and DE states^63,80–84^. Therefore, in various previous research, the expression of *Mixl1*, *Eomes*, *GSC*, *Gata4/6*, *Sox17* and *Cxcr4* transcripts was used as crucial markers to confirm the ME and DE cells^76,77^. In this study, we analyzed the expression of these transcript markers to assess the ME and DE formation of both protocols. Our findings showed that the expression of ME markers, *Eomes* and *GSC*, was found the significant upregulation after treating Chir99021 for 24 h. The later generation of DE was defined by Activin A through the upregulated expression of *Sox17*, *Gata4*, *Gata6*. Together, these results demonstrated that Chir99021 synergizes Activin A to effectively generate DE cells from cAD-MSCs.

Next, DE cells from previous step were induced into PE cells. Importantly, PE progenitors represent a critical step of in vitro IPC differentiation^85,86^. In natural pancreatic development, it is required the development of DE into primitive gut, posterior foregut, and PE precursors, respectively^85–87^. Our study found that the combination of small molecules in PE induction medium, which consisted of taurine, retinoic acid (RA), FGF2, EGF, SB431542, dorsomorphin, nicotinamide (NIC), and DAPT, show the positive outcome, especially P.2.3. Here, the group treated with taurine alone revealed the hasty differentiation by the greater expression of endocrine percussor markers (*Nkx2.2*, *Pax4*), however, they showed the lowest expression of *Pdx1* and *Nkx6.1*, pancreatic endoderm and multipotent progenitor markers, which could cause the undesired commitments of the cell’s fates^88,89^. It is noticed that EGF, epidermal growth factor, could promote the proliferation of *Sox9*/*Pdx1*-positive pancreatic progenitors^90^. We found that the using of EGF in early pancreatic differentiation (P.2.3) resulted the most efficient induction of Ngn3-positive PE precursors, while the using of EGF in late pancreatic differentiation stage (P.2.2) confined the differentiation of Ngn3-positive PE cells. Previous study reported that the presence of EGF was able to repress the differentiation of PE cells^91^. In several research on vertebrates, the important role of RA in pancreatic developments were detailly described^92,93^, while FGF2, a factor of notochord, is required to initiate for pancreatic development by inhibiting Shh expression^94–96^. Our result also defines the role of RA and FGF2 in the early pancreatic development by showed the increased expression of the primitive gut and posterior foregut markers (*Hnf1b*, *Hnf4a*, *Hnf6*) and pancreatic endoderm marker (*Pdx1*). Combination of RA, dorsomorphin, and SB431542 achieved the effective commitment on Pdx1-positive cell induction^75^. Also, another previous study showed that the cocktail including EGF, BMP inhibitor, and NIC boosted the induction of *Nkx6.1*, another crucial regulator of pancreatic islet development^97^. DAPT, known as an inhibitor of Notch signaling pathway, directly represses the expression of *Hes1* (Notch targeted gene)^98^*.* Transcription of *Hes1* gene inhibits the promotion of *Ngn3* gene^99^, while *Ngn3* expression is essentially required for endocrine cell development^98^. Our previous studies proved that the inhibition of Notch signaling during PE induction benefits IPC generation from MSCs^38,69^. Although RA could also suppress the *Hes1* expression indirectly^93^, further analyses are still required. BMP antagonism dorsomorphin inhibited Smad1/5/8 phosphorylation to expand the levels of *Ins* expression, which affected the production of essential hormone “Insulin” in β-cell development process^100,101^. Moreover, the inhibitor of TGFβ type I receptor, SB431542, also promotes the increased the expression of *Ins*^101^. Interestingly, our discovery revealed that the upregulation of *Sox9*, *Nkx6.1*, *Pax4*, *NeuroD1*, *Isl1*, and *Ngn3* was found by combined treatment with RA, SB431542, dorsomorphin, NIC, and DAPT. Furthermore, our study also found that a modified medium P.2.3 restricted the expression of *Cdkn1a*, a cell cycle regulator, during PE induction process. *Cdkn1a* was known that it plays a critical role in the cellular mediation by the overexpression of *Cdkn1a* results in cell cycle arrest^102^. Therefore, the suppression of this marker means the proliferative ability of IPCs. However, the further evaluation is still required. In summary, cAD-MSC-derived PE progenitors could be enriched when cAD-MSC-derived DE cells were treated with the combination of taurine, RA, FGF2, and EGF for 2 days, and then the mixture of taurine, RA, SB431542, dorsomorphin, NIC, and DAPT for 3 days.

Regarding the natural pancreatic development, PE cells can give rise to all types of pancreatic islets (alpha, beta, delta, epsilon, and upsilon)^85–87^. Therefore, the generation of islet β-like cells, which can secrete insulin responding to glucose stimulation, is the aim of in vitro IPC induction^33,85–87^. Unfortunately, cAD-MSC-derived PE colonies tend to lose their shape and decrease their number after the PE induction process (not shown data), this problem was also found in a previous study^103^. Meanwhile, the entrapment of 3D-PE organoids not only maintains their shapes, but also improves their differentiation property and hormone synthesis^103–106^. Hence, cAD-MSC-derived PE colonies were encapsulated in alginate gel before they were induced to IPCs. In this study, the functional IPC generation was found when PE precursors were treated with the mixture of taurine, NIC, Glp-1, NEAA, forskolin, LY294002, and SB431542. Forskolin and Glp-1 play important roles in insulin releasing process via the membrane adenylate cyclase (AC) cascade to covert ATP to cAMP, then modulate insulin secretion via PKA and PKC pathway. Forskolin directly stimulates AC, while Glp-1 will stimulate after the binding to its receptor^107–109^. Moreover, the inhibition of SB431542 via the TGFβ/ALK5 pathway could induce the expression of *MafA* and *NeuroD1* because of the upregulation of Foxo1 protein^108^. The inhibition of phosphoinositide 3-kinases (PI3K)/AKT pathway, LY290042, also potentiates the functional maturation of IPCs^51,110^. Here, *MafA*, *Insulin*, and *Glucagon* were the essential markers for islet maturation^111^, while *Glut2, Epac2* and *Rfx6* were reported to increase the IPC-related hormone secretion capacity^107–109,112,113^. Interestingly, we found that the expression of *MafA*, *Insulin*, *Glucagon*, *Glut2, Epac2* and *Rfx6* was upregulated in cAD-MSC-derived IPCs treated with P.3.2 (the combination of taurine, NIC, Glp-1, NEAA, forskolin, LY294002, and SB431542). Subsequently, the trend of concentration-dependent response upon 22 mM glucose stimulation was also discovered. Moreover, the higher expression of *Ngn3* was found on day 13 compared to PE at day 8, thus this cocktail, named “P.3.2” could stimulate the production of PE precursors alongside the mature IPCs. Hence, the IPCs on day 13 contained not only mature IPCs but also *Ngn3*-expressing PE cells, the similar results were also found in earlier studies^114–116^. These evidences indicate that cAD-MSC-derived PE can be more likely to undergo differentiation into IPCs by treatment with the combination of taurine, NIC, Glp-1, NEAA, forskolin, LY294002, and SB431542.

According to our previous study, although cAD-MSCs showed as the potential MSC candidate for IPC generation from a simple three-stepwise induction protocol and low attachment culture technique, the trend of glucose-responsive C-peptide secretion upon high (22 mM) glucose stimulation was not found different compared to normal (5.5 mM) glucose condition^38^. In this study, we improved the three-stepwise induction protocol through establishing the new microenvironment manipulation of each step. Interestingly, our modified induction protocol showed the improvement of cAD-MSCs-derived IPC generation. The trend of concentration-dependent response upon high (22 mM) glucose was notably superior compared to basal and normal (5.5 mM) conditions which reflects the glucose sensing of generated IPCs. Therefore, IPCs from the modified protocol may show sufficient function for improving hyperglycemic state in vivo*.*

The entrapment of IPCs in alginate not only maintains 3D-floating colony shape, but also enhances differentiation and hormone synthesis^103–106^. Besides, the aims of encapsulation are to immobilize the implants as well as build a wall from the body’s immune system for in vivo application^117^. Several previous studies were reported that the encapsulation of pancreatic islet, IPCs from ESCs and iPSCs using alginate could maintain the viability and functionality both in vivo and in vitro^53,104,105,118–120^. However, a greatest obstacle of monolayer capsule is the protrusion of cells. Our previous study showed that ALGPA-encapsulated hDPSC-derived IPCs can be preserved their viability and functionality^24^. Thus, ALGPA encapsulation may solve protrusion of IPCs and create favorable conditions for clinical treatments. In this study, we hypothesized that the in vitro preservation of ALGPA-encapsulated cAD-MSC-derived IPCs can provide the availability of IPCs for in vivo application requirements.

Here, three media were employed to investigate the preservation of ALGPA-encapsulated cAD-MSC-derived IPCs; M.4.1 (normal DMEM), M.4.2, and M.4.3 (VSCBIC-1). The specialized medium, VSCBIC-1, could maintain the morphology, viability, and functionality of mouse islets^29^. In addition, VSCBIC-1 could resuscitate the impaired islets derived from gut leak-induced IL-10 knockout mice^29^. Excitingly, the result showed that VSCBIC-1 could help to preserve viability of the outer cells in colonies after 13 days of IPC induction. In contrast, we found mass of non-viable cells in the core of encapsulated IPC colonies that was correlated to our previous study^24^. Additionally, we analyzed the expression of *Cdkn1a* which could activate anti-apoptosis and response to DNA damage or metabolic stress for supporting the cell survival^121,122^, and the better upregulation of *Cdkn1a* was found in VSCBIC-1.

Moreover, VSCBIC-1 improved functionality of ALGPA-encapsulated cAD-MSC-derived IPCs. Interestingly, the maintained upregulation of *Ngn3* was also found in VSCBIC-1. Likewise, it has been reported that taurine, an essential amino acid, affects the pancreas development, enhances and maintains the endocrine function, and increases the size and number of the islets^123^. Also, NIC, a form of vitamin B, acts as a poly (ADP-ribose) polymerase inhibitor that is used to promote MSCs homing functional PE/IPCs and preserve the islet viability and function by protection of NAD^+^/NADH ratio^117,124^. Thus, both taurine and NIC are used during differentiation into PE/IPCs and in vitro preservation. Which both small molecules were contained in both M.4.2 and M.4.3. On other hand, our observations suggested that the long-term induction with forskolin, LY294002, and SB431542, resulted in over-maturation of cAD-MSCs-derived IPCs as the extremely higher expression of markers in regard to IPC’s maturation, the lower expression of *Ngn3* and the tremendously highest C-peptide secretion were detected on day 27 post-induction.

Briefly, VSCBIC-1 medium preserves the colonies in both PE and premature/mature IPC statuses until day 14 since the last day of the IPC induction, while the IPCs in P.4.2 medium were seemingly found in mature IPC status. Additionally, the colony population in VSCBIC-1, which contains both PE and premature/mature IPCs, is suitable for transplantation with the following reasons. First, in vitro generated PE/premature IPCs would get more maturation after transplantation^125–130^. Second, PE cells could be able to take a longer than mature IPCs for achieving the reversed hyperglycemia in DM patients due to the prolonged maturation period in vivo^129,131–133^. Third, downregulation of *Ki67* from IPCs in VSCBIC-1 indicated that the proliferation is inhibited, this might be safe for transplantation because of restricted invasion capacity^134–136^. Fourth, the ability on releasing insulin/C-peptide upon high glucose stimulation was still responded when they were maintained in VSCBIC-1. Moreover, a lower expression of HLA on hESC-derived PE/premature IPCs permits maintenance of immune privilege^137^, this also indicated that PE/premature IPCs are more suitable for transplantation. Taken together, the potential of VSCBIC-1 medium is not only in preservation of IPCs, but also in production of RTU cAD-MSC-derive IPCs.

## Conclusion

Briefly, we established the three-stepwise protocol for generating IPCs from cAD-MSCs for 13 days. Combined management of small molecules could induce proficient differentiation into DE, PE precursors and IPCs. Our findings suggested that cAD-MSC-derived IPCs could be well-generated with our modified protocol. Moreover, these present results approved that the viability and functionality of ALGPA-encapsulated cAD-MSC-derived IPCs can be preserved for 14 days in specialized medium VSCBIC-1. For the further study, in vivo study is required to evaluate the safety and potential of clinical application of ALGPA-encapsulated cAD-MSC-derived IPCs.

## Materials and methods

### cAD-MSC isolation and culture

All procedures were conducted in compliance with the ARRIVE guidelines and according to regulations approved by the Institutional Animal Care and Use Committee (IACUC), Faculty of Veterinary Science, Chulalongkorn University (Animal Use Protocol No. 1531072). According to the inclusion criteria, four healthy dogs aged from 10 months to 5 years old were recruited (all dogs weighted over 5 kg) with their owner’s consent. Each of cAD-MSC line, which was collected from each donor dog (n = 4), was used in this study. Briefly, biopsied adipose tissues were collected from abdominal fat after anesthesia by veterinarians at Small Animal Hospital, Chulalongkorn University. cAD-MSCs were isolated following our previous study^38^, and they were reserved in the cell inventory for this study. In brief, adipose explants were washed twice with Phosphate Buffer Saline (PBS, Thermo Fisher Scientific Corporation, USA) before they minced and incubated in Cell Recovery Solution (Corning, USA) for 2 h at 37 °C. Then, the mixture was passed through the Falcon 70 µm Cell Strainer (Corning). Cells were collected by centrifuge at 2000 rpm for 5 min, and then resuspended and cultured onto T-75 flasks (Corning) contained Dulbecco’s Modified Eagle Medium (DMEM, Thermo Fisher Scientific) under 37 °C, 5% CO_2_ in humidified environment. The medium was supplemented with 10% Fetal Bovine Serum (FBS, Thermo Fisher Scientific), 1% GlutaMAX™ (Thermo Fisher Scientific), and 1% Antibiotic–Antimycotic (Thermo Fisher Scientific). Cells were subcultured once 80% confluence was reached. Cells at passage 3–5 were used for all experiments.

### Characterization and multilineage differentiation potential assay

cAD-MSCs were characterized by cell morphology under a phase-contrast microscope, and mRNA expression relating to stemness markers (*Oct4* and *Rex1*) and a proliferation marker (*Ki67*) by RT-qPCR. Besides, MSC-related surface markers were analyzed by flow cytometry. In particular, the cells were stained with FITC-conjugated mouse anti-human CD45 antibody (BioLegend, USA), mouse anti-human CD73 monoclonal antibody (Thermo Fisher Scientific) and FITC-conjugated goat anti-mouse immunoglobulin G (IgG) secondary antibody (BioRad, USA), PE-conjugated rat anti-dog CD90 monoclonal antibody (eBioscience, USA), Alexa Fluor 488-conjugated rat anti-dog CD44 antibody (BioRad), PE-conjugated mouse anti-human CD29 monoclonal antibody (Bio Legend). FITC-conjugated mouse IgG1 kappa Isotype (BioLegend), mouse IgG2a kappa Isotype (Thermo Fisher Scientific), PE-conjugated rat IgG2b kappa Isotype (eBioscience), Alexa Fluor 488-conjugated rat IgG2a Isotype (BioRad), PE-conjugated mouse IgG1 kappa Isotype (BioLegend) were used as isotype controls. The results were analyzed using a FACScalibur flow cytometer with CellQuest software (BD Bioscience, USA).

Differentiation potentials of cAD-MSCs were investigated using adipogenic, osteogenic, and chondrogenic induction protocols according to our previous study^41,69,138^.

For adipogenic differentiation, 3 × 10^4^ cAD-MSCs were seeded onto a 24-well culture plate and treated with adipogenic induction medium supplemented with 10% FBS, 0.1 mg/mL insulin (Sigma), 1 μM dexamethasone, 1 mM 3-isobutyl-1-methylxanthine (IBMX, Sigma), and 0.2 mM indomethacin (Sigma) for 28 days^41,69,138^. Afterwards, the intracellular lipid droplets were detected with Oil Red O (Sigma) staining and the expression of adipogenic mRNA markers (*Leptin*, and *LPL*) was assessed by RT-qPCR.

For osteogenic differentiation, cAD-MSCs (2.5 × 10^5^ cells/well) were seeded onto 24-well culture plate (Corning) and induced with osteogenic induction medium contained DMEM supplemented with 10% FBS, 50 mg/mL ascorbic acid (Sigma, USA), 100 mM dexamethasone (Sigma), and 10 mM β-glycerophosphate (Sigma) for 14 days^69^. Extracellular matrix (ECM) mineralization was detected using Alizarin Red S (Sigma) dye. Osteogenic mRNA markers (*Runx2* and *Ocn*) were determined by RT-qPCR.

For chondrogenic differentiation, cAD-MSCs were induced in chondrogenic induction medium comprised of 15% FBS, 0.1 μM dexamethasone, 50 mg/mL L-ascorbic-2–2-phosphate (AA2P, Sigma), 4 mg/mL L-proline (Sigma), 2% antibiotic–antimycotic, and 10 ng/mL transforming growth factor (TGF)-β3 (Sigma), and 1% insulin-transferrin-selenium (ITS, Thermo Fisher Scientific) for 21 days^41,69,138^. Subsequently, glycosaminoglycan formation was detected by Alcian Blue (Sigma) staining and the expression of chondrogenic mRNA markers (*Sox9* and *Col2a1*) was evaluated by RT-qPCR.

### In vitro insulin-producing cell differentiation

The protocol of insulin-producing cell (IPC) induction was separated into three main stages including definitive endoderm (DE), pancreatic endocrine progenitors (PEP), and insulin-producing cells (IPCs). All induction media were using DMEM as the basal media which were not adding any FBS, called “serum-free media (SFM)”.

For DE induction, cells were collected by trypsinization and centrifuge at 2000 rpm, 4 °C for 5 min. Then, a million of cells was induced into DE with 2 different protocols. In P.1.1, the cells were cultured in SFM-1.1 supplemented with 1% ITS, 1% BSA (Cohn fraction V, fatty acid free, Sigma), 4 nM Activin A (Sigma), and 1 nM sodium butyrate (Sigma) for 3 days. In P.1.2, the cells were cultured SFM-1.2 supplemented with DMEM supplemented with 1% BSA, 4 nM Chir99021 (Sigma), and 1 nM sodium butyrate for a day before changing to SFM-1.1 for 2 days.

For PE precursor induction, DE clusters were induced into PE with 3 different protocols. In P.2.1, SFM-2.1 contained 1% BSA, 1% ITS, and 0.3 mM taurine (Sigma) was utilized for 5 days. In P.2.2, DE clusters were cultured in SFM-2.2.1 supplemented with 1% BSA, 1% ITS, 0.3 mM taurine, and 20 ng/mL bFGF (Sigma) for 2 days, then the medium was changed to SFM-2.2.2 which was supplemented with 1% BSA, 1% ITS, 0.3 mM taurine, 2 µL retinoic acid (RA, Sigma), 10 mM nicotinamide (Sigma), 25 µL DAPT (Sigma), 1 µL dorsomorphin (Sigma), 10 µL SB431542 (Sigma) and 50 ng/mL EGF (Sigma) for 3 days. In P.2.3, DE clusters were culture in SFM-2.3.1 supplemented with 1% BSA, 1% ITS, 0.3 mM taurine, 20 ng/mL bFGF and 50 ng/mL EGF for 2 days, then the medium was replaced by SFM-2.3.2 contained 1% BSA, 1% ITS, 0.3 mM taurine, 2 µM RA, 10 mM nicotinamide, 25 µL DAPT, 1 µM dorsomorphin, and 10 µM SB431542, and cultured for 3 days.

When the PE induction protocol was verified, PE cells were continually differentiated toward IPCs after they were encapsulated with alginate gel (Sigma). Two different protocols were utilized to induce PE cells to IPCs for 5 days. P.3.1 was made by DMEM supplemented with 1% BSA, 1% ITS, 3 mM taurine, 1 mM nicotinamide, 100 nM glucagon-like peptide-1 (GLP-1, Sigma), and 1% non-essential amino acids (NEAA, Sigma). P.3.2 was supplemented 1% BSA, 1% ITS, 3 mM taurine, 1 mM nicotinamide, 100 nM GLP-1, 1% NEAA, 10 µM SB431542, 10 µL forskolin (Sigma), and 10 µM LY294002 (Sigma).

### Encapsulation

The protocol of colony encapsulation was followed by a previous report^24^, cAD-MSC-derived colonies were harvested and resuspended in 2% alginate solution. Sterile polystyrene syringe and 22G needle (Nipro, Japan) were used to generate alginate beads. Drops of beads were collected in 100 mM CaCl_2_ (Sigma-Aldrich) under stirring conditions, and then washed by Krebs–Ringer- Hepes (KRH) containing CaCl_2_ buffer. For double-layer encapsulation, a cold solution of 30% pluronic F127 was added to cover all surfaces of alginate beads at room temperature (RT).

### Maintenance of the alginate/Pluronic acid-encapsulated IPC’s function in vitro

Subsequently of IPC induction, three different media were applied to consider the maintenance ability of the IPCs’ function and viability in vitro. M.4.1 is basic DMEM with 10% FBS. M.4.2 is an IPC induction medium (P.3.2). M.4.3 is VSCBIC-1 which was prepared following our previous study^29^.

### Reverse transcription quantitative polymerase chain reaction (RT-qPCR)

The total cellular RNA was extracted using TRIzol™ reagent (Thermo Fisher Scientific) and Direct-zol™ RNA Miniprep kit (Zymo Research, USA) according to the manufacturer’s protocol. Successively, the cDNA was obtained from RNA using ImProm-II™ Reverse Transcription System (Promega, USA). Targeted genes were amplified and detected by FastStart Essential DNA Green Master (Roche Diagnostics, USA) and CF96™ real-time PCR detection system (BioRad). The mRNA expression was illustrated as relative mRNA expression by normalized to Glyceraldehyde 3-phosphate dehydrogenase (*Gapdh*) and the undifferentiated cells as a control. The 2^−∆∆t^ formula was used to calculate relative gene expression. All primers were designed by NCBI primer designing tool based on the mRNA sequences from NBCI database (https://www.ncbi.nlm.nih.gov). The primers sequences and their accession number are shown in Supplementary Table S1.

### Immunocytochemistry (ICC)

IPC colonies were fixed in cold methanol for 15 min at RT, then permeabilized with 0.1% Triton-X100 (Sigma). After that, the background was blocked with 10% donkey serum for 1 h. The primary antibodies, rabbit anti-insulin (Cell signature technology, USA)^139^ at dilution 1:200 and mouse anti-rat glucagon (Abcam, USA)^140^ at dilution 1:200, were added and incubated overnight. Then, cyanine (Cy) 3-conjugated donkey anti-rabbit IgG (Bio Legend) and FITC-conjugated goat anti-mouse IgG (BioRad) were used as secondary antibodies, respectively. After incubation with secondary antibodies for 2 h, DAPI was used to stain the nucleus. The results and images were acquired using a fluorescent microscope ZEISS Apotome.2 (Carl Zeiss, Germany) incorporated with Axio Observer Z1 and ZEN pro software (ZEISS International, Germany).

### Functional test and enzyme-linked immunosorbent assay (ELISA)

The IPCs were assessed the function by glucose-stimulated C-peptide secretion assay (GSCS)^24,38,69,70,138^. The IPCs were incubated in normal KRH bicarbonate (KRBH) buffer (pH 7.4) for 1 h as basal control, then in 5.5 mM of glucose anhydrous (Sigma) in KRBH for the next 1 h and finally in 22 mM glucose anhydrous in KRBH for 1 h. Enzyme-linked immunosorbent assay (ELISA) kit (Millipore) was used for detecting the generated C-peptide level according to the manufacturer’s protocol. Total DNA (ng) and stimulation time (mins) were used to normalization.

### Live/dead staining

Encapsulated IPCs were evaluated for their viability using the NUCLEAR-ID Blue/Red cell viability reagent (GFP-CERTIFIED) (Enzo Life Science, USA), according to the manufacturer’s protocol. The result was clarified under a fluorescent microscope (ZEISS Apotome.2 (Carl Zeiss, Germany) incorporated with Axio Observer Z1 and ZEN pro software (ZEISS International, Germany).

### Statistical analyses

The total number of colony and their size distribution were determined using ImageJ software and standardized with hemocytometer square size from 10 randomly positions. The results were illustrated by a dot plot (n = 4) using GraphPad Prism 9.0 (Graph Software Inc., San Diego, CA). SPSS Statistics 22 software (IBM Corporation, USA) was employed to analyze the results. The Mann–Whitney U test was exercised to compare two independent groups, while the Kruskal–Wallis was devoted to analyze three or more experimental groups. Statistically, a significant difference was considered as a *p*-value < 0.05.

## Supplementary Information

Supplementary Information.

## Data Availability

The RT-qPCR gene expression data has been deposited to the Gene Expression Omnibus (GEO) repository under accession number GSE196118. The other generated and analyzed datasets during the current study are available from the corresponding author upon reasonable request.
