# Supplementary material for: In vitro generation of transplantable insulin-producing cells from canine adipose-derived mesenchymal stem cells
Source: Sci Rep. 2022 Jun 1;12:9127. doi: 10.1038/s41598-022-13114-3 (PMC9160001; doi:10.1038/s41598-022-13114-3)
Supplement: Supplementary file 1 — Supplementary Information. [file 41598_2022_13114_MOESM1_ESM.pdf]

Supplementary information

In vitro generation of transplantable insulin-producing cells from canine adipose-derived mesenchymal stem cells

Quynh Dang Le[1,2,4], Watchareewan Rodprasert[2], Suryo Kuncorojakti[2,3], Prasit Pavasant[6], Thanaphum Osathanon[6,7], Chenphop Sawangmake[2,4,5,8*]

[1] International Program of Veterinary Science and Technology, Faculty of Veterinary Science, Chulalongkorn University, Bangkok, Thailand

[2] Veterinary Stem Cell and Bioengineering Innovation Center (VSCBIC), Veterinary Pharmacology and Stem Cell Research Laboratory, Faculty of Veterinary Science, Chulalongkorn University, Bangkok, Thailand

[3] Department of Veterinary Science, Faculty of Veterinary Medicine, Universitas Airlangga, Surabaya – East Java, Indonesia

[4] Veterinary Stem Cell and Bioengineering Research Unit, Faculty of Veterinary Science, Chulalongkorn University, Bangkok, Thailand

[5] Department of Pharmacology, Faculty of Veterinary Science, Chulalongkorn University, Bangkok, Thailand

[6] Department of Anatomy and Center of Excellence for Regenerative Dentistry (CERD), Faculty of Dentistry, Chulalongkorn University, Bangkok, Thailand

[7] Dental Stem Cell Biology Research Unit, Faculty of Dentistry, Chulalongkorn University, Bangkok, Thailand

[8] Center of Excellence in Regenerative Dentistry, Faculty of Dentistry, Chulalongkorn University, Bangkok, Thailand

* Corresponding authors

E-mail: chenphop.s@chula.ac.th

SUPPLEMENTARY TABLE

Supplementary Table S1. Primers sequences

Genes	Accession number	Sequences	5'-3'	Length (bp)	Tm (ºC)
**Stemness markers**					
Oct4	XM_538830.1	Forward<br>Reverse	AGGAGAAGCTGGAGCAAAACC<br>GTGATCCTCTTCTGCTTCAGGA	100	60.55<br>59.50
Rex1	XM_003639567.1	Forward<br>Reverse	AGGTTCTCACAGCAAGCTCA<br>CCAGCAAATTCTGCGCACTG	199	59.24<br>60.73
**Proliferation marker**					
Ki67	XM_014108788.1	Forward<br>Reverse	GTGCAACTAAAGCACGGAGA<br>GAGATTCCTGTTTGCGTTTTCGT	124	58.49<br>58.49
**Cell cycle regulator**					
Cdkn1a	XM_532125.6	Forward<br>Reverse	GACTTTCCCCACTGCCCTAC<br>GCCCTATCCACAGCGTCTAC	108	60.04<br>59.97
**Osteogenic markers**					
Runx2	XM_005642335.1	Forward<br>Reverse	GGAAGAGGCAAGAGTTTCACC<br>GTGCTCACTTGCCAACAGAA	209	58.84<br>58.89
OCN	XM_547536.4	Forward<br>Reverse	GCCAGCCTATGGTCTCCTCTG<br>CCACCAGCTCCTTCTGTTCTCT	249	61.90<br>54.55
**Adipogenic makers**					
Leptin	NM_001003070.1	Forward<br>Reverse	TGTGGCTTTGGCCCTATCTG<br>CAGCGACCCTCTGTTTGGAG	147	60.03<br>60.67
LPL	XM_005635734.3	Forward<br>Reverse	CTGGAGAGACTCAGAAAAAGGT<br>AAT<br>TCCTTCTGTAGATTTGCTCAGGT	148	58.29<br>59.16
**Chondrogenic markers**					
Sox2	XM_005639752.2	Forward<br>Reverse	AGAAGGATAAGTACACGCTGCC<br>TTCATGTGCGCGTAGCTGTC	130	60.16<br>61.35
Col2a1I	NM_001006951.1	Forward<br>Reverse	ATGAAAGACTGCCTCAGCCC<br>TCTGTCCCTTTGGTCCTGGT	103	60.03<br>60.40
**Mesendodermal markers**					
Mixl1	XM_022417967.1	Forward<br>Reverse	ACCCCTCAGTCCCCTGATTT<br>TCAGGGAGCTGGTGGTATGA	120	60.18<br>59.96
Eomes	XM_005634337.3	Forward<br>Reverse	CAGTCGAGAAGGGCAGAAAG<br>GGAGGTTGACCGAAAAAGCA	161	58.28<br>58.69
GSC	XM_005623927.2	Forward<br>Reverse	TGGAACAAGACCTCGTCCAAG<br>TCTTTGTCCCCTGACCATCG	135	59.93<br>59.39
**Definitive endoderm markers**					
Gata4	NM_001048112.1	Forward<br>Reverse	TCCCCACAAGGCTACACATC<br>ATGCAGTGATTATGTCCCCGT	112	59.38<br>59.51
Gata6	XM_022421725.1	Forward<br>Reverse	TACAGCAAGATGAACGGCCT<br>GGTTCACCCTCCGCATTTCT	140	59.39<br>60.32
Cxcr4	NM_001048026.1	Forward<br>Reverse	GTTGAGGCTGTGGCAAACTG<br>GTAGACCACCTTTTCCGCCA	186	59.97<br>59.96
Sox17	XM_544084.5	Forward<br>Reverse	GGGGGAGAAAAATGTGGGGT<br>GGGTTCTGACAACGCAACTG	75	59.88<br>59.69
**Pancreatic endoderm marker**					

Pdx1	NM_001284471.2	Forward<br>Reverse	AAGTCTACCAAGGCTCACGC<br>GTGCCTCTCGGTCAAGTTCA	201	60.04<br>59.97
**Primitive gut and posterior foregut markers**					
Hnf1β	XM_005624790.3	Forward<br>Reverse	TGCTATTGAACTGAGCCACACA<br>AAGGGGAATGGAGGGGCAAT	170	61.22<br>61.23
Hnf6	XM_846134.5	Forward<br>Reverse	CCCCAAACCCTGGAGCAAAC<br>TTGGGTGTGTTGCCTCTATCC	108	61.47<br>60.00
Hnf4a	XM_022408885.1	Forward<br>Reverse	GCGACCTGCTGTTTGATGGT<br>GGAGGCGTGATAGCCAGATAG	105	61.24<br>59.80
**Multipotent pancreatic progenitor markers**					
Ptf1a	XM_845293.4	Forward<br>Reverse	GAACAGCCAAAGTGTGGACC<br>ACTCAAAGGGCGGTTCGTTT	90	59.33<br>60.46
Sox9	NM_001002978	Forward<br>Reverse	CAGCACAAGAAAGACCACCC<br>GAAATGTGCGTCTGTTCGGT	107	59.05<br>59.13
Nkx6.1	XM_544960.5	Forward<br>Reverse	CAGGAGTTATGCAGAGCCCG<br>ACGTGGGTCTCGTGTGTTTT	111	60.53<br>60.11
**caNotch pathway-related gene**					
Hes1	XM_025478075.1	Forward<br>Reverse	GAGAAGGCGGACATTCTGGA<br>ACCTCGTTCATACACTCGCTG	137	59.46<br>60.14
**Pancreatic endocrine markers**					
Nkx2.2	XM_542867.6	Forward<br>Reverse	GACACCAACGACGAGGAAGG<br>AGCGCGTATATGGGTTGTCG	169	60.67<br>60.60
Pax4	XM_022427277.1	Forward<br>Reverse	GCCCCTTAGACCTTGCTCTG<br>CTGCTTGGGCAGGATTAGGT	114	60.11<br>59.74
Ngn3	XM_546140.1	Forward<br>Reverse	TCTGAGCAAGCAGCGACG<br>AGCGCCCAGATGTAGTTGTG	177	60.43<br>60.39
NeuroD1	XM_005640377.3	Forward<br>Reverse	AGTTCGAGAAGAGCTACGCC<br>CTCGTGATGCGAATGGCTCT	155	59.55<br>60.53
Isl1	XM_848628.4	Forward<br>Reverse	TGGCTTACAGGCAAACCCAG<br>GACATCGACGCCACTTCACT	171	60.54<br>60.39
Glut2	XM_545289.5	Forward<br>Reverse	ACTCATCACAGGACGTGGAG<br>AGCTGAGTGTAGCGGTGAAG	108	59.11<br>59.76
**Pre-mature endocrine marker**					
MafB	XM_005635059	Forward<br>Reverse	TGCCCAGTGGTGACCTAAAC<br>AAGGACGCGCTTGAAAGTTG	105	59.89<br>59.69
**Mature pancreatic endocrine markers**					
Glp1-r	XM_014118246.1	Forward<br>Reverse	CACGGTGGGCTATACACTCTC<br>AGGACGCAAACAGGTTCAGG	116	59.93<br>60.54
MafA	XM_003431814.3	Forward<br>Reverse	GCTTCAGCAAGGAGGAGGTC<br>CTCTGGAGCTGGCACTTCTC	136	60.39<br>60.11
Glis3	XM_022421745.1	Forward<br>Reverse	CAGGTCTTGTAGGCCTTGGG<br>GCTGGTCGTGGACATCAAAC	109	60.04<br>59.48
Insulin	NM_001130093.1	Forward<br>Reverse	CGGCTTCTTCTACACGCCTA<br>GCGCCCCTAGTTGCAGTAAT	202	59.55<br>60.46
Glucagon	NM_001003044.1	Forward<br>Reverse	TCCAATCGCGGTGTCAGAAG<br>ACCCTGAGAATGACGCTTGT	197	60.39<br>59.31
**Hormone release-related markers**					
Rfx6	XM_022419390.1	Forward	TGTTTAGGGCACAGCCTCAC	154	60.25

		Reverse	TAGCTGGAAGGTGGTCGAGA		59.96
Epac2	XM_014110689.1	Forward	GAACGATCCAGTGAAGATGTGG	174	59.07
		Reverse	GCCAGGACAGCATACCAGTT		60.04
**Reference marker**					
Gapdh	NM_001003142.1	Forward	CCAACTGCTTGGCTCCTCTA	100	59.38
		Reverse	GTCTTCTGGGTGGCAGTGAT		59.67
